# A plasma metabolomic signature discloses human breast cancer

**DOI:** 10.18632/oncotarget.14521

**Published:** 2017-01-05

**Authors:** Mariona Jové, Ricardo Collado, José Luís Quiles, Mari-Carmen Ramírez-Tortosa, Joaquim Sol, Maria Ruiz-Sanjuan, Mónica Fernandez, Capilla de la Torre Cabrera, Cesar Ramírez-Tortosa, Sergio Granados-Principal, Pedro Sánchez-Rovira, Reinald Pamplona

**Affiliations:** ^1^ Department of Experimental Medicine, University of Lleida-Institute for Research in Biomedicine of Lleida (UdL-IRBLleida), Lleida, Spain; ^2^ Department of Oncology, Medical Oncology Unit, Hospital San Pedro de Alcántara, Cáceres, Official Postgraduate Programme in Nutrition and Food Technology, University of Granada, Spain; ^3^ Institute of Nutrition and Food Technology “José Mataix”, Biomedical Research Center, Department of Physiology, University of Granada, Granada, Spain; ^4^ Institute of Nutrition and Food Technology “José Mataix”, Biomedical Research Center, Department of Biochemistry and Molecular Biology II, University of Granada, Granada, Spain; ^5^ Department of Medical Oncology, Hospital of Jaén, Jaén, Spain; ^6^ Department of Pathological Anatomy, Hospital of Jaén, Jaén, Spain; ^7^ GENYO, Centre for Genomics and Oncological Research (Pfizer / University of Granada / Andalusian Regional Government), PTS Granada, Granada, Spain

**Keywords:** breast cancer, biomarker, mass spectrometry, metabolites, metabolomics

## Abstract

**Purpose:**

Metabolomics is the comprehensive global study of metabolites in biological samples. In this retrospective pilot study we explored whether serum metabolomic profile can discriminate the presence of human breast cancer irrespective of the cancer subtype.

**Methods:**

Plasma samples were analyzed from healthy women (n = 20) and patients with breast cancer after diagnosis (n = 91) using a liquid chromatography-mass spectrometry platform. Multivariate statistics and a Random Forest (RF) classifier were used to create a metabolomics panel for the diagnosis of human breast cancer.

**Results:**

Metabolomics correctly distinguished between breast cancer patients and healthy control subjects. In the RF supervised class prediction analysis comparing breast cancer and healthy control groups, RF accurately classified 100% both samples of the breast cancer patients and healthy controls. So, the class error for both group in and the out-of-bag error were 0. We also found 1269 metabolites with different concentration in plasma from healthy controls and cancer patients; and basing on exact mass, retention time and isotopic distribution we identified 35 metabolites. These metabolites mostly support cell growth by providing energy and building stones for the synthesis of essential biomolecules, and function as signal transduction molecules. The collective results of RF, significance testing, and false discovery rate analysis identified several metabolites that were strongly associated with breast cancer.

**Conclusions:**

In breast cancer a metabolomics signature of cancer exists and can be detected in patient plasma irrespectively of the breast cancer type.

## INTRODUCTION

There is a close relationship between metabolism and cancer. Cancer cell metabolism undergoes a profound rearrangement featured by changes in metabolic networks mostly involved in bioenergetic and biosynthetic processes [1]. This metabolic switch represents an adaption to support cell survival, tumor growth, tissue remodeling, and cancer metastasis. But whereas available evidence suggest that this metabolic adaption is regulated by a genomic program and influenced by the tumor microenvironment, in some circumstances altered metabolism can play a primary role in oncogenesis [1, 2]. Furthermore, metabolism can also determine the course of the cancerous process or even lead to an adverse drug response.

Breast cancer is the most common malignancy and cause of cancer death in women [3, 4]. Common methods for diagnosis and surveillance include mammography, histopathology and blood tests (such as antigens and protein patterns). Since the success for curative intervention and significantly increase long-term survival rates in breast cancer is in early stage disease, more sensitive biomarkers for early detection and molecular targets for better treating breast cancer are needed.

In this setting new profiling tools provide a global picture of tumor biology including development and progression. The comprehensive analysis of metabolites (‘metabolomics’), by high-resolution ^1^H nuclear magnetic resonance (NMR) spectroscopy and mass spectrometry (MS), are being currently used to identify and define the metabolic phenotype of subcellular organelles, cell types, or tissues. These metabolomics approaches are providing key information about oncogenesis, uncovering potential new therapeutic targets and will be a key tool in cancer diagnosis [1, 5, 6].

The human plasma metabolome is composed of around 4,229 confirmed compounds that can be grouped into more than 50 chemical classes [7]. Plasma metabolome profile is the result of a homeostatic system that expresses, in a bidirectional interaction, cellular needs and specific physiological cell-tissue states. Consequently, cell-tissue cancer could modify the chemical composition of blood plasma/serum, analogously to the association of specific metabolomics signatures with complex biological processes such as aging and diseases such as Alzheimer's disease, cardiovascular disease and metabolic disorders [8–11]. So, a potential strength of plasma metabolomic analysis is that this approach can provides a composite metabolomic snapshot of both the tumor and the host.

Since breast cancer displays a high heterogeneity from histology to prognosis, metastatic evolution and treatment responses, and in view of the need for more refined diagnosis estimation in breast cancer, we designed this study to explore whether metabolomics can add diagnosis information in individuals with breast cancer. We assessed plasma metabolomic profiles in newly diagnosed breast cancer patients using a liquid chromatography-mass spectrometry (LC-ESI-QTOF MS/MS) platform-based metabolomics approach, with the hypothesis that in breast cancer a metabolomics signature of cancer exists and can be detected in patient plasma irrespectively of the breast cancer type.

## RESULTS

### Metabolomics profiling in plasma by LC-ESI-QTOF MS/MS in breast cancer and healthy groups

**Figure 1:**
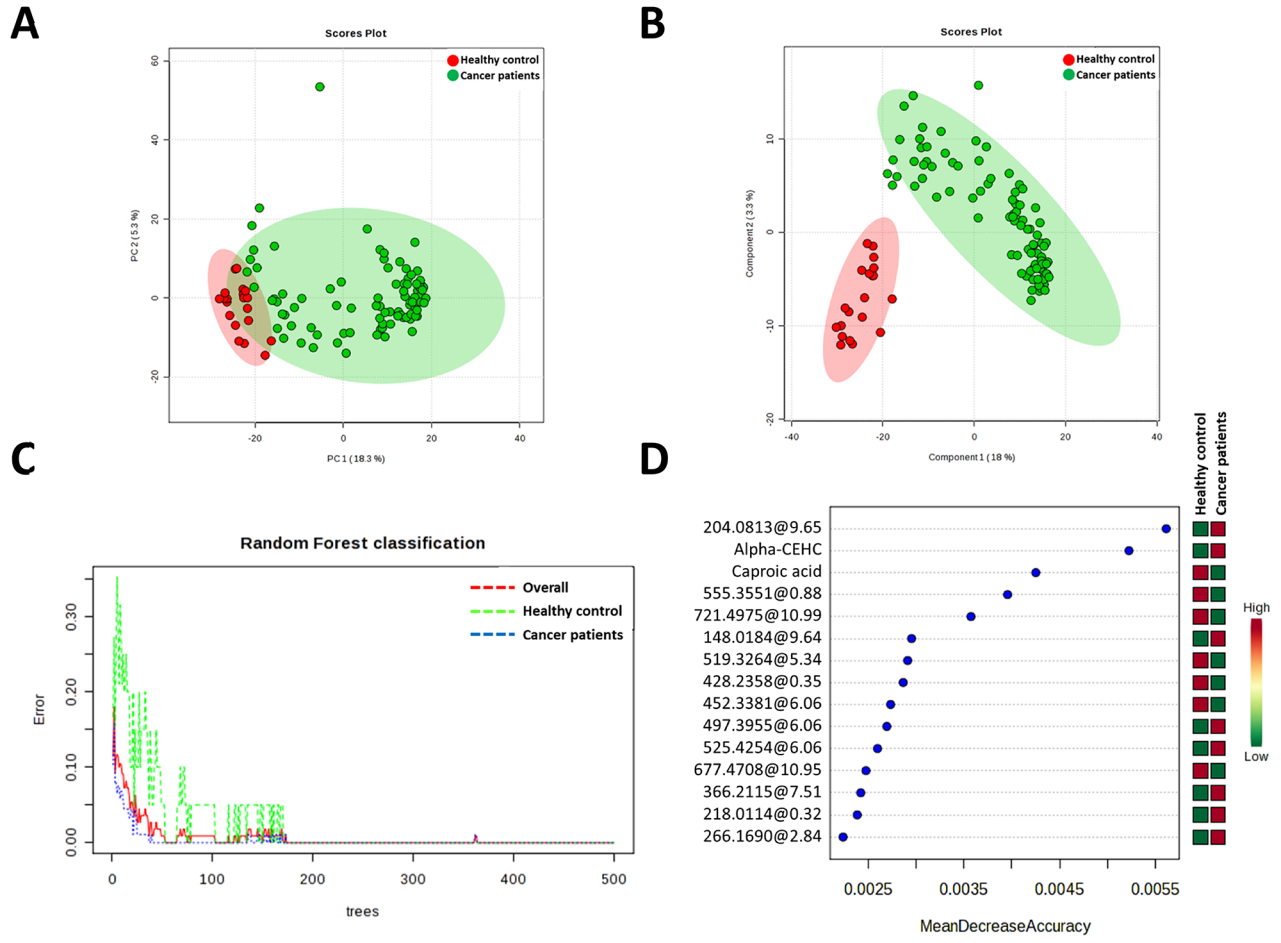
Multivariate analyses reveals specific metabolomic signature of cancer patients plasma samples **A**. Principal Component Analyses revealed a good clusteritzation of samples from cancer group. **B**. Partial Least Discriminating analysis define a perfect metabolic signature for both groups. **C**. Random Forest classification shows and overall classification error of 0 (0 for healthy and cancer patient groups). **D**. Metabolites which much contribute to Random Forest classification. Unknown identities are represented as exact mass@retention time.

The first aim of this work was to analyze global metabolomic differences between breast cancer and healthy samples. To do this, we applied a non-targeted metabolomics approach focusing on the profiles of low molecular weight (m/z < 1500) ionizable molecules which were present in at least 50% of the samples of each group (2356). To determine whether the metabolite fingerprints in fasting plasma differed between breast cancer and healthy control subjects in our metabolomics approach, we first evaluated separation between experimental groups using unsupervised principal component analyses (PCA) (Figure 1A). Strong group separation was achieved in plasma between all two groups, suggesting the existence of a specific metabolomic signature for each condition. Further analysis using partial least square discriminant analysis (PLS-DA) models demonstrated robust group separation between both groups (Figure 1B) obtaining good cross validation results (Max components= 5; C-V method= 10-fold CV; Performance measure= Q2) (Supplementary Table 1).

Multivariate classification analyses were comple-mented applying Random Forest (RF) analyses, a supervised class prediction model, in order to a) determine the capacity for global metabolomes to accurately classify patients into their respective groups and b) to identify metabolites most important to the class prediction and hence which possessed the strongest correlation to the respective disease. In the RF supervised class prediction analysis comparing breast cancer and healthy control groups, RF accurately classified 100% both samples of the breast cancer patients and healthy controls (Figure 1C). So, the class error for both group in and the out-of-bag error were 0. The metabolites which major contribute to classification were shown in Figure 1D.

### Altered metabolites and canonical pathways in plasma of breast cancer patients and healthy control subjects

After multivariate statistics analyses we applied a Student's T Test (p<0.05, Benjamini-Hochberg False Discovery Rate) to define which metabolites were statistically altered in breast cancer patients. We found 1269 metabolites with different concentration in plasma from healthy controls and cancer patients (Supplementary DataSet). Basing on exact mass, retention time and isotopic distribution we could identify 35 metabolites (Table 1) belonging to aminoacyl-tRNA biosynthesis, arginine and proline metabolism and primary bile acid biosynthesis pathways (Table 2), among others.

**Table 1:** Metabolites statistically significant (p<0.05, Benjamini-Hoghberg False Discovery Rate) with a potential identity

Compound	p (Corr)	Regulation (cancer patients vs healthy control)	FC	Mass	Retention Time
2-Hydroxy-3-methylbutyric acid	3.3E-04	up	36.6	100.0522	1.5816069
2-Hydroxy-3-methylpentanoic acid	2.3E-02	up	3.3	114.0682	3.2419913
2-Methylhippuric acid	1.8E-03	down	−92.0	175.0639	3.6565607
2-Octenoic acid	5.3E-03	down	−39.3	372.268	9.767441
3-Hydroxyanthranilic acid	8.8E-06	down	−341.8	135.0336	1.5612222
3-Methylglutaric acid	5.5E-03	up	37.6	128.0489	1.5807501
4-acetamidobutanoate	2.0E-03	down	−53.3	127.064	0.70647365
5-b-Cholestane-3a, 7a, 12a-triol	3.6E-02	down	−13.2	402.3519	12.439621
5α-androstane-3,17-dione	1.2E-10	down	−148.8	305.2386	10.385417
7-ketocholesterol	4.3E-04	up	117.0	400.3335	12.138314
7α-hydroxy-cholesterol	8.9E-03	down	−31.4	384.329	12.368574
Caproic acid	8.4E-17	down	−1.7	348.2573	5.769
Chenodeoxycholic Acid	6.1E-04	up	161.8	392.2908	11.337122
Cortisol	3.1E-05	down	−1.7	362.2124	7.031
Cortisone	3.0E-02	down	−5.8	360.1945	6.9761095
Creatine	3.9E-04	down	−339.6	113.0561	0.42863637
Cytidine	3.5E-02	up	21.9	225.0778	0.7119473
DL-pipecolic acid	1.5E-06	up	270.4	129.0792	0.33790255
Dopamine	7.8E-04	up	1.5	135.0675	0.575
Glutamine	2.0E-06	down	−1060.4	146.0684	0.5461304
Hippuric acid	3.8E-02	down	−8.8	179.0599	2.1277783
Homocystine	2.9E-04	up	62.3	306.0068	0.33542165
Inosine diphosphate (IDP)	1.8E-03	up	52.7	410.0028	0.34574685
L-Arginine	1.5E-05	down	−397.2	174.1067	0.4356315
Linoleic acid	4.1E-17	up	42496.8	280.2411	11.370296
L-Lysine	1.7E-04	down	−61.4	146.1059	0.34584
L-Valine	1.7E-02	down	−64.5	117.0775	0.44549397
Myristic acid	2.7E-04	up	78.9	250.1932	12.096725
N-Oleoyl-D-erythro-Sphingosine (C18:1 Ceramide)	7.2E-07	down	−645.7	571.51	13.315624
Oleamide	3.8E-05	up	2.0	281.2726	11.383955
Retinoic acid	1.3E-08	down	−128.9	863.6179	11.356807
Stearic acid	2.0E-06	up	673.6	284.2717	12.056651
Taurine	6.4E-09	up	198.1	125.0153	0.32860422
Threonate	3.9E-02	up	3.9	136.0378	0.88052344
Uric acid	2.8E-02	up	2.3	168.0292	0.6809543

**Table 2:** Pathways modulated by breast cancer condition

Pathway name	Total	Expected	Hits	p
Aminoacyl-tRNA biosynthesis	75	0.87246	4	0.010095
Arginine and proline metabolism	77	0.89572	4	0.011061
Primary bile acid biosynthesis	47	0.54674	3	0.01624
Nitrogen metabolism	39	0.45368	2	0.074242
Purine metabolism	92	1.0702	3	0.088937
D-Arginine and D-ornithine metabolism	8	0.093062	1	0.089485
Lysine degradation	47	0.54674	2	0.10237
Fatty acid biosynthesis	49	0.57	2	0.10982
Biotin metabolism	11	0.12796	1	0.12101
D-Glutamine and D-glutamate metabolism	11	0.12796	1	0.12101
Pyrimidine metabolism	60	0.69796	2	0.15306
Linoleic acid metabolism	15	0.17449	1	0.16141
Taurine and hypotaurine metabolism	20	0.23265	1	0.20939
Retinol metabolism	22	0.25592	1	0.22783
Alanine, aspartate and glutamate metabolism	24	0.27919	1	0.24586
Pantothenate and CoA biosynthesis	27	0.31408	1	0.27215
Valine, leucine and isoleucine biosynthesis	27	0.31408	1	0.27215
Lysine biosynthesis	32	0.37225	1	0.314
Steroid hormone biosynthesis	99	1.1516	2	0.32119
Propanoate metabolism	35	0.40715	1	0.33799
Valine, leucine and isoleucine degradation	40	0.46531	1	0.37618
Ascorbate and aldarate metabolism	45	0.52347	1	0.41224
Phenylalanine metabolism	45	0.52347	1	0.41224
Fructose and mannose metabolism	48	0.55837	1	0.43291
Glycine, serine and threonine metabolism	48	0.55837	1	0.43291
Cysteine and methionine metabolism	56	0.65143	1	0.48465
Tyrosine metabolism	76	0.88409	1	0.59485
Tryptophan metabolism	79	0.91899	1	0.60928

**Figure 2:**
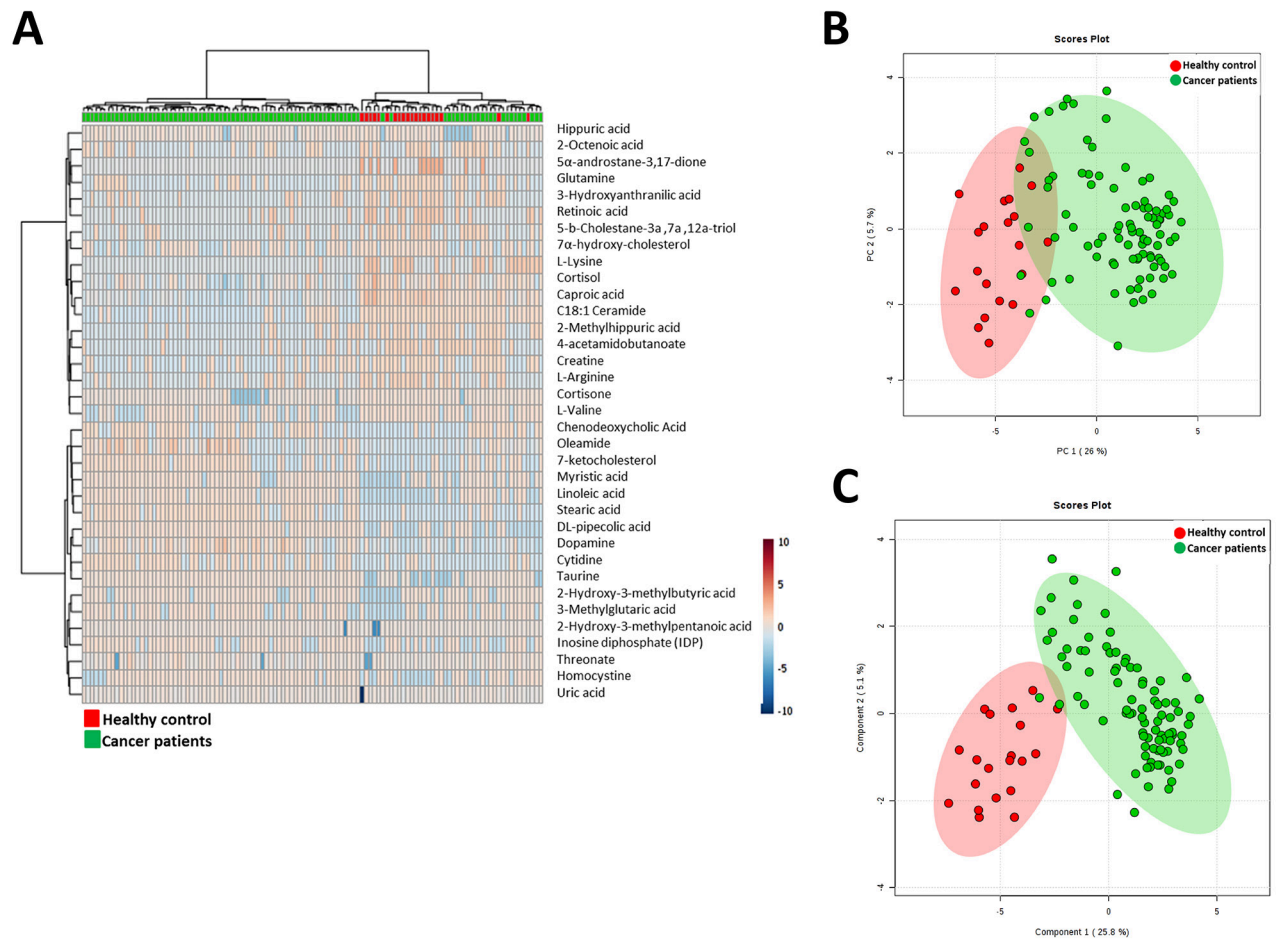
**A**. Hierarchical clustering analyses using the statistical significant metabolites which has a potential identity (based on exact mass, retention time and isotopic distribution. **B**. Principal Component Analyses performed with the statistical significant metabolites which has a potential identity (based on exact mass, retention time and isotopic distribution. **C**. Partial Least Square Discriminant Analysis performed with the statistical significant metabolites which has a potential identity (based on exact mass, retention time and isotopic distribution.

To further analyze whether these molecules could define the metabolic status of cancer patients we performed a multivariate statistics using only these molecules which present a statistically significant difference between groups and have a potential identity (based on exact mass, retention time and isotopic distribution) (Figure 2). First of all, we applied hierarchical analyses were we could see relative concentration of each metabolite (Figure 2A). This analysis also shows a good clusteritzation of samples from cancer patients. In the same line, both PCA and PLS-DA analyses showed that, although the separation is better using all molecules detected, we could define a signature using only 35 metabolites (Figure 2B and 2C). Both permutation test (Supplementary Figure 2) and cross-validation results (Max components= 5; C-V method= 10-fold CV; Performance measure= Q2) (Supplementary Table 2) validate PLS-DA model. Finally, in order to control overfitting we used an alternative technique for multivariate analyses, the RF analyses obtaining an out-of-bag error of 0.027 (Supplementary Figure 2). Overall, these results supports an specific metabolomic signature using only 35 molecules.

### Receiver operator characteristic (ROC) curve analysis

**Figure 3:**
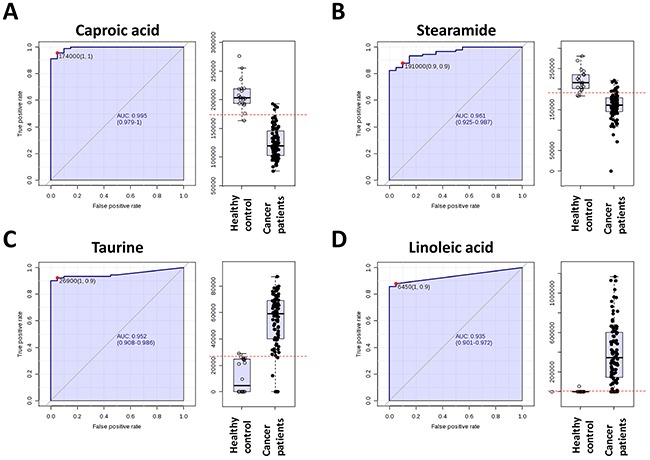
Receiver operating characteristic curve of caproic acid, stearamide, taurine and linoleic acid

The collective results of RF, significance testing, and false discovery rate analysis identified several metabolites that were strongly associated with breast cancer. To further characterize the predictive value of these metabolites to discriminate breast cancer, we performed ROC analysis using MS peak areas (Table 3). We found 3 metabolites (metabolite 1: 542.2335@6.062038 (p=3.2109E-18), metabolite 2: 497.3955@6.065792 (p=2.6216E-14), metabolite 3: 204.0813@9.653965 (p=5.7445E-38)) with an area under the curve (AUC) = 1, a specificity= 1 and a sensibility = 1. Among the metabolites with a putative identity we found with highest significant the caproic acid (AUC = 0.995, specificity= 1 and a sensibility = 1), the taurine (AUC = 0.952, specificity= 0.9 and a sensibility = 1), staramide (AUC = 0.959, specificity= 0.9 and a sensibility = 0.9) and the linoleic acid (AUC = 0.935, specificity= 0.9 and a sensibility = 1) (Figure 3).

**Table 3:** Receiver operator characteristic (ROC) analysis of metabolites significantly associated with the presence of breast cancer

Metabolite	Accurate mass@ retention time	Sensitivity	Specificity	AUC	p	Fold difference in breast cancer vs. healthy controls
C26H43ClN4S3	542.2335@6.062	100	100	1.00	3.21e-18	0.98
C26H51N5O4	497.3955@6.065	100	100	1.00	2.62e-14	1.32
C9H16O3S	204.0813@9.653	100	100	1.00	5.74e-38	1.08
C23H30N2S	366.2115@7.516	100	100	0.999	4.76e-17	2.08
278.1552@9.641	278.1552@9.641	100	100	0.999	6.15e-36	1.06
Caproic acid	348.2573@5.769	100	100	0.995	4.12e-23	0.99
Taurine	125.0153@0.328	100	90	0.952	3.048e-14	0.66
Stearamide	283.2877@11.795	90	90	0.959	2.3782e-12	0.85
Linoleic Acid	280.2411@11.37	100	90	0.935	8.7246e-8	6.29

Metabolites investigated through ROC analysis were selected on the basis of their value to Random Forest, p-value and false discovery rate, and fold difference in breast cancer vs. healthy controls. Mass spectrometry peak areas corresponding to expression level in each patient were used in the ROC analysis.

## DISCUSSION

Breast cancer has been associated with marked metabolic shifts [2] [12–19] [20–34]. Since now, metabolomics has been mainly used to refine molecular subtyping of breast cancer, cancer progression, cancer metastasis, and prediction of treatment sensitivity. Only a few metabolomics breast cancer studies have been conducted in plasma/serum mostly focused to discriminate breast cancer subtypes [35], metastatic breast cancer [36–41], recurrence [42, 43] and response to neoadjuvant chemotherapy [44].

The present study demonstrate for the first time that a metabolic signature of breast cancer exists and can be detected in patient plasma. Thus, we found 1269 metabolites with different concentration in plasma from healthy controls and cancer patients. Among them, 354 could be identified (based on exact mass, retention time and isotopic distribution) and different functions could be attributed. Specifically, some of the metabolites could be involved in cell growth by providing building stones for the synthesis of essential cellular components, and substrates for bioenergetics. So, the lower plasma concentrations of the amino acids valine, arginine, tryptophan and lysine in breast cancer patients could express the higher uptake of these amino acids by the tumor, but also a preferential utilization of them. In addition, the elevated content in taurine and homocysteine is also suggestive of increased utilization of the amino acid methionine, essential for the synthesis of methyl group donor compounds, the amino acid cysteine, and the antioxidant glutathione [45]. In this line, the higher content of linoleic acid and stearic acid, as well as cytidine (also used for phosphatidylcholine and phosphatidylethanolamine biosynthesis) [46], suggest a higher rate of structural lipids biosynthesis. Furthermore, the higher plasma concentration of cytidine (pyrimidine nucleoside), inosine diphosphate (purine nucleoside) and uric acid suggest increased need of substrates for nucleic acid biosynthesis by the tumor. In parallel, the elevated content in short- and medium-chain fatty acids (caproic acid, and myristic acid), the lower content in glutamine and creatine, and higher content of taurine, suggest increased bioenergetics of tumor cells.

In this context it is also particularly interesting the detection of increased levels in breast cancer patients of three metabolites belonging to the branched chain amino acid (BCAA) metabolism (2-hydroxy-3-methylbutiric acid, 2-hydroxy-3-methylpentanoic acid, and 3-methylglutaric acid) suggesting that BCAA are preferentially used by breast cancer cells likely to provide carbon for gluconeogenesis. Because i) BCAAs have a central role in the maintenance of lean body mass and regulation of skeletal muscle protein metabolism [47] and ii) cancer cachexia is characterized by increased oxidation of BCAAs, and net catabolism of skeletal muscle through a reduction in protein synthesis and activation of proteolysis, it is postulated that breast cancer activates metabolic pathways which induce cachexia.

Other metabolites which show antioxidant activity (taurine and uric acid) were increased in plasma from cancer subjects could be involved in protecting cancer cells from excessive damage by oxidative stress. Reinforcing this fact, a lower concentration of the oxidative stress-derived compounds 7alpha-hydroxy-cholesterol and 3-hydroxyanthranilic acid (oxidation product of tryptophan) were detected in the breast cancer group.

Finally, among differential metabolites endogenous signaling lipids were found. Thus, we detected a decreased content of retinoic acid, C18:1 ceramide and two N-acyl amino acids (2-methylhippuric acid and hippuric acid), while the endocannabinoid oleamide is increased in breast cancer group. Globally, all these changes seem to be designed to enhance cell proliferation and tumor cell survival.

In summary, the changes described in the metabolomic profile in breast cancer patients may affect disease biology in different ways. Specifically, these metabolites may promote tumorigenesis by changing the differentiation status of tumors, induce metastatic phenotype, or make tumors more viable in oxidative stress conditions. But in any case, metabolomics studies in human plasma from breast cancer patients could be useful to describe diagnostic and/or prognosis biomarkers, as well as for monitoring treatment.

## MATERIALS AND METHODS

### Participants and ethics

A total of 91 breast cancer patients and 20 healthy control subjects were recruited at the Breast Cancer Medicine Service at Hospital of Jaén (Jaén, Spain). The study was approved by the institutional review board of the Clinical Research Ethics Committee of the Hospital of Jaén, and every patient provided written informed consent for participation. The criteria for selection included: at last 18 years old with histological confirmation of breast cancer; no detectable macrometastatic disease, and no prior anticancer treatment. Demographic characteristics and clinical diagnosis of studied subjects are summarized in Table 4. In order to avoid the effect of potential cofounders (such as age, BMI, menopause, diabetes, cholesterol and drug treatment) in metabolomics analyses the homogeneity of both groups was checked. We applied Student T-test for continuous variables (age, BM and cholesterol) and Fisher's exact Test for two way categorical data (menopause, diabetes and drug treatment). Among cofounders analyzed only BMI presents statistically significance (p=0.0057) between groups. To further analyze the effect of BMI in plasma metabolomics profile we performed multivariate statistics which showed that BMI, contrary to pathology, did not have any effect in determining plasma metabolomic profile (Supplementary Figure 1). Further, one-way ANOVA on BMI (Normal Weight (BMI: 18.5-24.9); Overweight (BMI: 25-29.9); Obese (BMI>30)) showed no statistically significant metabolites between groups.

**Table 4:** Demographic and clinical pathological characteristics of study population

	Breast cancer patients	Healthy control subjects
Biospecimen	Plasma	Plasma
Number of participants	91	20
Age (median, range)	62 (34-91)	48 (22-64)
TNM stage-I	2 (2.1%)	n.a.
TNM stage-IIa	40 (43.4%)	n.a.
TNM stage-IIb	30 (32.6%)	
TNM stage-IIIa	13 (14.1%)	n.a.
TNM stage-IIIb	7 (7.6%)	
TNM stage-IV	0	n.a.
Luminal A	25 (27.1%)	n.a.
Luminal B	38 (41.3%)	n.a.
HER-2	25 (27.1)	n.a.
Triple Negative	12 (13.0%)	n.a.

Abbreviations: n.a., not applicable.

Samples were collected in EDTA tubes at 08:00 hours in the morning after at least 8h of fasting using standard venipuncture procedures. Blood was processed by centrifugation within 2 h of collection using a gradient of histopaque in order to separate plasma, erythrocytes and PBMC. Plasma samples were isolated, aliquoted and stored at -80°C until further use.

### Sample processing

Metabolites from plasma were extracted as previously described [9]. Samples were thawed on ice at 4°C, and 300 μl of cold methanol (containing 1 μM of hutylhydroxytoluene as antioxidant and 1 μg/ml of ^13^C-phenylalanine as internal standard) were added to 100 μl of plasma for deproteinization, followed by incubation at -20°C for 1h and then, centrifuged at 12000g for 3 min. The supernatants were recovered, evaporated using a Speed Vac (Thermo Fisher Scientific, Barcelona, Spain) and re-suspended in water 0.4% acetic acid/methanol (50/50).

### Metabolomic analyses

For the metabolomic study, an Agilent 1290 LC system coupled to an ESI-Q-TOF MS/MS 6520 instrument (Agilent Technologies) was used. In all cases, 2 μL of extracted sample was applied onto a reversed-phase column (Zorbax SB-Aq 1.8 μm 2.1 × 50 mm; Agilent Technologies) equipped with a precolumn (Zorba-SB-C8 Rapid Resolution Cartridge 2.1 × 30 mm 3.5 μm; Agilent Technologies) with a column temperature of 60°C. The flow rate was 0.6 mL/min. Solvent A was composed of water containing 0.2% acetic acid and solvent B was composed of methanol 0.2% acetic acid. The gradient started at 2% B and increased to 98% B in 13 min and held at 98% B for 6 min. Post-time was established in 5 min.

Data were collected in positive electrospray mode time of flight operated in full-scan mode at 100–3000 m/z in an extended dynamic range (2 GHz), using N_2_ as the nebulizer gas (5 L/min, 350°C). The capillary voltage was 3500 V with a scan rate of 1 scan/s. The ESI source used a separate nebulizer for the continuous, low-level (10 L/min) introduction of reference mass compounds: 121.050873, 922.009798 (positive ion mode) and 119.036320, 966.000725 (negative ion mode), which were used for continuous, online mass calibration. MassHunter Data Analysis Software (Agilent Technologies, Barcelona, Spain) was used to collect the results, and MassHunter Qualitative Analysis Software (Agilent Technologies, Barcelona, Spain) to obtain the molecular features of the samples, representing different, co-migrating ionic species of a given molecular entity using the Molecular Feature Extractor algorithm (Agilent Technologies, Barcelona, Spain), as described [9, 48]. Finally, MassHunter Mass Profiler Professional Software (Agilent Technologies, Barcelona, Spain) and Metaboanalyst platform [49] were used to perform a non-targeted metabolomic analysis of the extracted features. We selected samples with a minimum of 2 ions. Multiple charge states were not considered. Compounds from different samples were aligned using a retention time window of 0.1% ± 0.25 minutes and a mass window of 10.0 ppm ±2.0 mDa. Only common features (found in at least 50% of the samples of any group) were analyzed, correcting for individual bias. PCA, PLS-DA, RF analyses, Hierarchical analyses and ROC curves were done using Metboanalyst platform [49]. Then, we applied univariate statistics (Student's T test, p<0.05, Benjamini-Hochberg false discovery rate) evaluate significant differences induced by carcinogenic process. The resulting differential metabolites were searched against PCDL database from Agilent (Agilent Technologies, Barcelona, Spain), which uses retention times in a standardized chromatographic system as an orthogonal searchable parameter to complement accurate mass data (accurate mass retention time approach) according to previously published works [48]. Pathway analysis was performed using Metaboanalyst platform [49].

### Abbreviations

AUC, area under the curve; BCAA, branched chain amino acids; MS, mass spectrometry; RF, random forest; ROC, receiver operating curves; PCA, principal component analyses; PLS-DA, partial least square discriminant analysis

## SUPPLEMENTARY MATERIALS FIGURES AND TABLES


