# Supplementary material for: A plasma metabolomic signature discloses human breast cancer
Source: Oncotarget. 2017 Jan 5;8(12):19522–33. doi: 10.18632/oncotarget.14521 (PMC5386702; doi:10.18632/oncotarget.14521)
Supplement: Supplementary file 1 [file oncotarget-08-19522-s001.pdf]

A plasma metabolomic signature discloses human breast cancer

SUPPLEMENTARY FIGURES AND TABLES

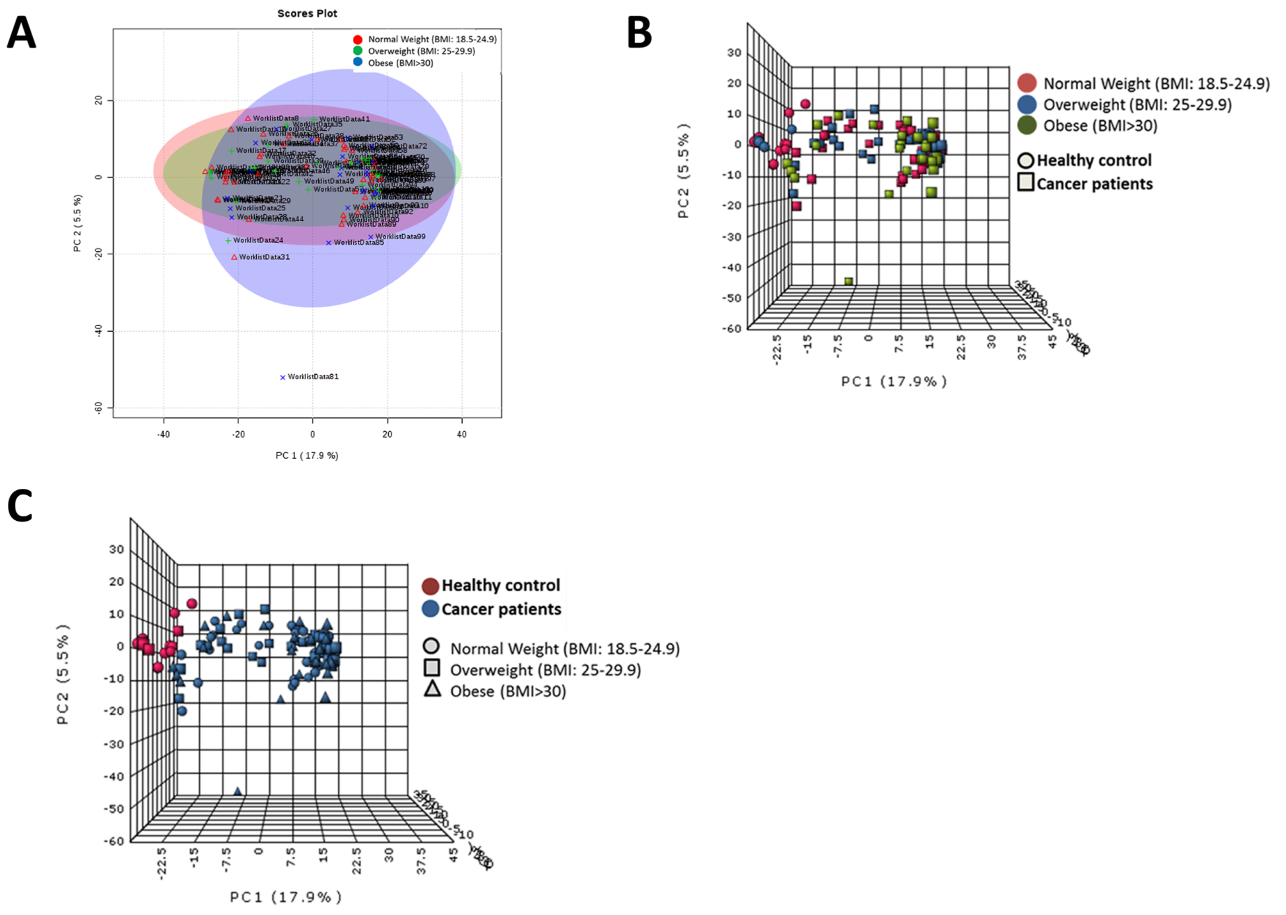

Supplementary Figure 1: BMI variable do not determine a plasma specific metabolomic signature. A. Principal component representation indicates that BMI is not an important factor describing metabolome in these samples. Specifically, cancer condition determines an specific metabolomic signature comparing with healthy controls, independently of BMI value of the subjects **B.** and **C**.

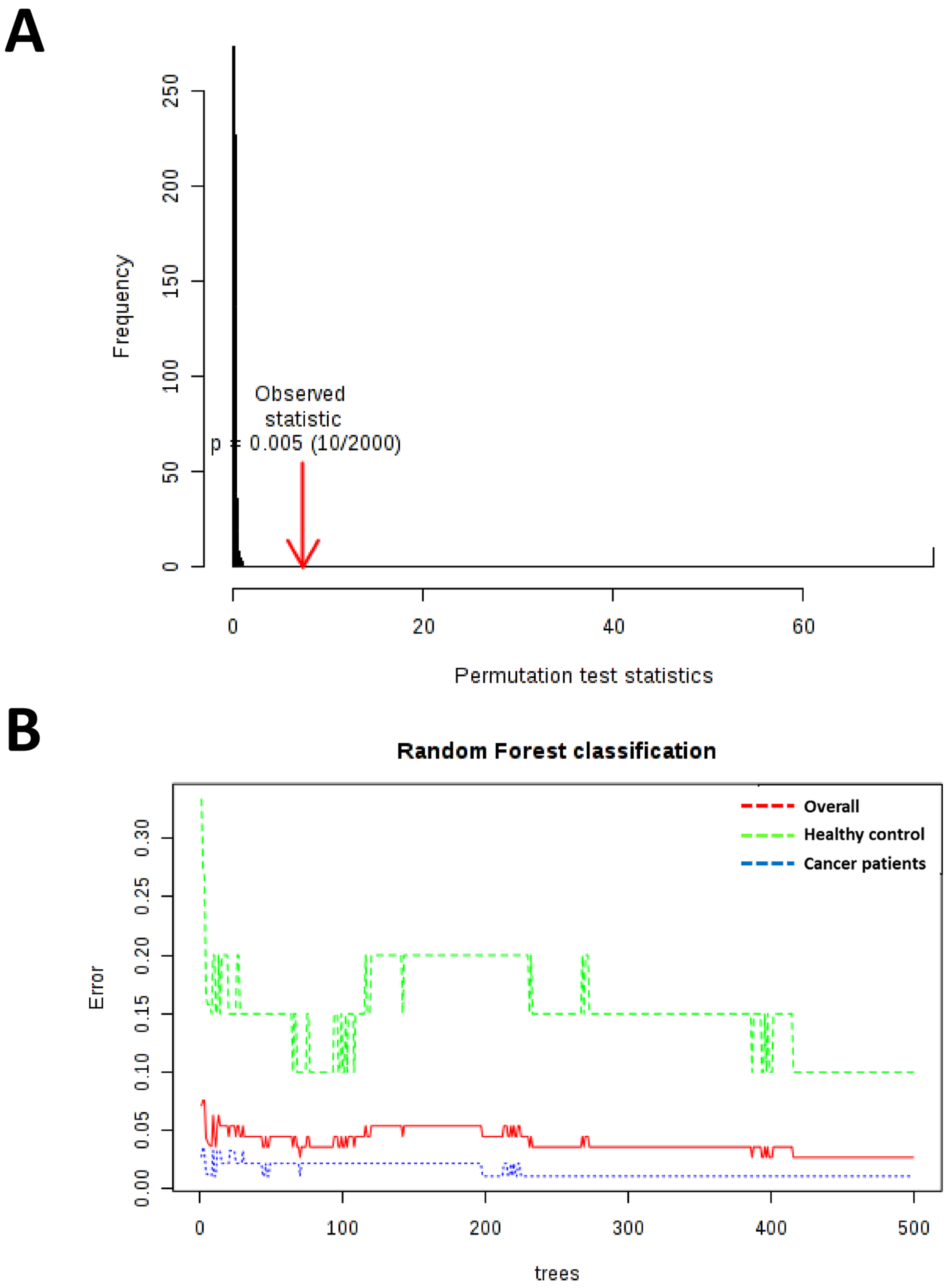

Supplementary Figure 2: A. Permutation test of PLS-DA using only the metabolites with a potential ID demonstrate that none of the distributions formed by the permuted data was better than the observed statistic based on the original data **B, C.** Random Forest classification shows and overall classification error of 0.027 using only that metabolites with a potential ID.

Supplementary Table 1: PLS-DA cross validation details (using those molecules present at least in 50% of the samples of the same group)

Measure	1 comps	2 comps	3 comps	4 comps	5 comps
Accuracy	0.948	1.0	0.98	0.97	0.98
R2	0.57875	0.87072	0.94092	0.97992	0.99615
Q2	0.49665	0.74071	0.77456	0.7795	0.77166

Supplementary Table 2: PLS-DA cross validation details (using those molecules which present a significant difference (p<0.05) and with a potential identity)

Measure	1 comps	2 comps	3 comps	4 comps	5 comps
Accuracy	0.97067	0.974	0.966	0.948	0.948
R2	0.64092	0.79248	0.81766	0.82385	0.82633
Q2	0.58961	0.69667	0.64811	0.6131	0.5879
